# Modulation of the Gut Microbiota and Liver Transcriptome by Red Yeast Rice and Monascus Pigment Fermented by Purple Monascus SHM1105 in Rats Fed with a High-Fat Diet

**DOI:** 10.3389/fphar.2020.599760

**Published:** 2021-01-21

**Authors:** Hua Yang, Ronghua Pan, Jing Wang, Lizhong Zheng, Zhenjing Li, Qingbin Guo, Changlu Wang

**Affiliations:** ^1^ State Key Laboratory of Food Nutrition and Safety, Ministry of Education, College of Food Science and Engineering, Tianjin University of Science and Technology, Tianjin, China; ^2^ Zhejiang Sanhe Bio-Tech Co., Ltd., Zhejiang, China

**Keywords:** red yeast rice, pigment, hyperlipidaemia, intestinal flora, RNA-seq

## Abstract

Hyperlipidemia can easily cause atherosclerosis and induce cardiovascular and cerebrovascular diseases. Red yeast rice (RYR) contains a variety of active ingredients and is commonly used as medicine and food, and has pharmacological effects such as lowering blood lipids. In this study, we select Monascus strain SHM1105 with a high yield of Monacolin K and monascus pigment (PIG), and studied the effects of the RYR and PIG fermented by this strain on blood lipids, intestinal flora, and liver transcriptome in hyperlipidemia model rats. The experimental results show that, compared with the high-fat model group, the weight growth rate, liver weight ratio, kidney weight ratio, spleen weight ratio, and fat weight ratio of rats in the gavage lovastatin (LOV), RYR, and PIG group were all significantly decreased (*p* < 0.05). Intervention with RYR and PIG can significantly reduce the serum TC, TG, and LDL-C levels, which has the effect of lowering blood lipids. The 16SrDNA sequencing results showed that the ratio of Firmicutes/Bacteroidetes decreased significantly (*p* ≤ 0.01) after the intervention of LOV, RYR, and PIG; the abundance of the ratio of *Lachnospiraceae*, *Ruminococcaceae*, *Prevotellaceae*, and *Bacteroidales-S24-7-group* also changed. The combined analysis of transcriptome and metabolome showed that lovastatin, RYR, and PIG can all improve lipid metabolism in rats by regulating Steroid hormone biosynthesis, Glycerolipid metabolism, and the Arachidonic acid metabolism pathway. In addition, RYR and PIG also have a unique way of regulating blood lipids. Although a lot of research on the lipid-lowering components of Monascus rice and the single pigment component of Monascus has been carried out, the actual application is RYR and pigments as mixtures, as a mixture of RYR and PIG contains a variety of biologically active ingredients, and each component may have a synergistic effect. Hence it has a lipid-lowering mechanism that lovastatin does not have. Therefore, RYR and PIG are effective in reducing lipid potential development and can be utilized in functional foods.

## Introduction

Obesity has become a global health problem and is closely related to the occurrence of fatty liver, hyperlipidemia, and cardiovascular and cerebrovascular diseases (Nakamura et al., 2014). Long-term high-fat and high-sugar diets can lead to obesity, cause hyperlipiddemia, and then promote the development of systemic dyslipidemia diseases such as Cardiovascular diseases (CVD) and Atherosclerosis (AS) (Teslovich et al., 2010; Jemil et al., 2017). The prevention and treatment of hyperlipidemia is a key measure to prevent and treat cardiovascular and cerebrovascular diseases and fatty liver syndrome. Drug and diet intervention can effectively reduce the risk of hyperlipidemia.

Lovastatin (LOV) is the earliest statin used clinically and reduces cholesterol levels by reversibly and competitively inhibiting 3-hydroxy-3-methyl-glutaryl coenzyme a reductase (HMG-Co-a) (Kuzma-Kuzniarska et al., 2015; Cui et al., 2016). Lovastatin has a direct effect on reducing total cholesterol (TC) and low-density lipoprotein cholesterol (LDL-C), thereby preventing the risk of coronary heart disease (Shattat, 2014; Faran et al., 2019). However, Lovastatin has certain side effects. Long-term use can cause adverse reactions, including liver function damage, muscle toxicity, and possible nervous system damage or tumor increase (Ali et al., 2017). Lovastatin has poor water solubility, a short half-life, and poor oral bioavailability (Zhou and Zhou, 2015). Studies have shown that treatment with lovastatin leads to slight impairments in neuropsychological tests of attention and psychomotor speed (Lovastatin. 2016). In the literature review, cases of interaction between erythromycin and lovastatin were also found, which manifested as rhabdomyolysis, elevated transaminase activity, and acute renal insufficiency (Lovastatin. 2016). However, red yeast rice (RYR), a functional food containing lovastatin, did not seem to cause these side effects.

RYR is a traditional medicine and food source in China and East Asia. It contains 25–73% sugars (starch in particular), 14–31% proteins, 2–7% water, and 1–5% fatty acids, sterols, isoflavones, and pigments such as rubropunctamine, monascorubramine, and polyketides (Ma et al., 2000; Patakova, 2013). Chen et al. studied the solubility, physical state, and oral bioavailability of RYR, and compared it with pure lovastatin. The results showed that monascus products had high solubility and reduced crystallinity, so they had higher and faster oral biodrug efficiency than pure lovastatin (Chen et al., 2013). In addition to lowering blood lipids, RYR also plays an important role in preventing cardiovascular and cerebrovascular diseases, and seems to be more safe (Li et al., 2009; Cicero et al., 2017; Cicero et al., 2019). Varady et al. used RYR extract in combination with octadecanol in 11 patients with normal/marginal triglyceridemia, moderate hypercholesterolemia, and low CVD risk. After two months of treatment, LDL-C levels were reduced by an average of 20% without any serious safety issues (Varady et al., 2003). In a randomized controlled study, patients with familial hypercholesterolemia were treated with RYR extract as a dietary supplement for eight weeks, and the treatment was determined to be effective, safe, and well tolerated, with those receiving the RYR combination showing an 18.5% reduction in total cholesterol and a 25.1% reduction in LDL-C (Abello et al., 2009). Even for patients who are intolerant to statins, RYR can have a good lipid-lowering effect and high safety (Marazzi et al., 2011). RYR combined with policosanols and berberine was found to be as effective as moderate-dose statins in lowering LDL-C in patients with primary hypercholesterolemia and a history of statin intolerance or refusal of statin treatment (Pisciotta et al., 2012). Thus, RYR seems to be an overall tolerable and safe lipid-lowering dietary supplement (Banach et al., 2018). Lovastatin used in clinical treatment has toxicity problems, however, RYR are natural molecules and are considerably less toxic (Shinkai, 2012; Rader and deGoma, 2014).

Monascus pigment (PIG) is a traditional natural functional pigment produced by the fermentation of monascus in China. It is applied to food coloring in the form of a mixture in practical applications and has high levels of safety (Chen et al., 2017). In addition to being used as a food additive, PIG also has the function of reducing fat. It has been shown that the orange pigment has anti-atherosclerosis effects and could be considered as a natural alternative used in clinical trials (Kim and Ku, 2018). Various pigment components, such as Anka monascus pigment, also have lipid-lowering effects, but the separation of various components is difficult. In practical applications, monascus pigments are often used in the form of mixtures, and multiple pigment components may have synergistic effects. However, the fat-reducing effect of PIG as a mixed pigment has not been evaluated, and the fat-reducing mechanism has not been studied.

Traditional studies on reducing blood lipid of Monascus and its fermentation products only focus on the role of a single component. In practical application, RYR and PIG are often used as mixtures in functional food or as food additives. Various components of natural products may have a comprehensive effect on the lipid-lowering effect. Therefore, it is very valuable to study the lipid-lowering mechanism of RYR and PIG as a whole.

Purple Monascus SHM1105 (CGMCC No.15369) is a high-yielding Monacolin K and pigment strain selected from red yeast rice. It has been applied to the production of functional RYR. It can be used as an experimental strain to study the fat-reducing mechanism of RYR and PIG. In this study, SD rats induced by a high-fat and high-sugar diet were used as an animal model to evaluate the lipid-lowering effects of RYR and PIG produced by the fermentation of purple Monascus SHM1105. Through the intestinal flora status, liver transcriptome, and metabonomic changes, the blood lipid-lowering mechanism of RYR and PIG produced by the fermentation of purple Monascus SHM1105 can also be studied. This may become the experimental basis for the application of RYR and PIG fermented by purple Monascus SHM1105 in the treatment and prevention of lipid metabolism diseases and the development of functional foods.

## Materials and Methods

### Reagents and Drugs

Monascus purpureus SHM1105 is deposited in the General Microbiology Center of China Microbial Culture Collection Management Committee, the deposit number is CGMCC No.15369, and the preservation date is March 21, 2018. TC kit, TG kit, LDL-C kit, HDL-C kit, ALT kit, and AST kit were purchased from Nanjing Jiancheng Institute of Bioengineering.

Preparation of RYR: Soak 150 g of rice in distilled water for 24 h, drain the water, steam for 20 min, inoculate 15 ml of Monascus seed solution, stir well, and incubate at 32°C for seven days.

Preparation of PIG: a) Extraction of pigment from mycelium: dry the fresh mycelium of Monascus after fermentation to a constant weight, grind it into powder with a mortar, pass through an 100-mesh sieve, and put it into a centrifuge tube. Add eight times the volume of 75% ethanol, undergo ultrasonic treatment for 30 min, 2,862 × g centrifugation for 10 min, and collect the supernatant in a volumetric flask; b) Pigment extraction in fermentation broth: put the fermentation broth in a centrifuge tube, add double the volume of 80% ethanol, and mix well. Heat in a 60°Cwater bath for 1 h, shake once every 10 min, centrifuge at 2,862 × g for 15 min, and take the supernatant. The monascus pigments extracted from two sources are mixed and freeze-dried to obtain the monascus pigments.

Seed solution preparation method: Seed solution culture medium: glucose 6 g, peptone 2 g, NaNO_3_ 1 g, MgSO_4_·7H_2_O 0.5 g, and KH_2_PO_4_ 1 g, dilute to 100 ml with tap water, adjust pH to about 5.5 with lactic acid, and sterilize at 121°C for 20 min. Add 5 ml of sterile water to the inclined surface of the preservation test tube, and gently scrape the spores with a sterile spore spatula to prepare a spore suspension. All the spore suspension was transferred to the seed liquid culture medium and cultured on a shaker at 30°C and 180 rpm for 36 h to prepare the seed liquid.

PIG fermentation method: inoculate the seed liquid into the pigment fermentation medium at a ratio of 3%, and ferment for eight days. Fermentation medium: 5 g rice flour, 0.3 g NaNO_3_, 0.1 g MgSO4·7H_2_O, 0.15 g KH_2_PO_4_, and 100 ml tap water, natural pH, and sterilize at 121°C for 20 min.

### Animals and Experimental Scheme

For the experiment, 50 healthy SD rats without specific pathogens [male, body weight (180 ± 20 g)] were chosen and purchased from Beijing Sibeifu Experimental Animal Co., Ltd. [license number: SCXK (Shanghai) 2012-0002]. All animal procedures were performed in accordance with the Guidelines for Care and Use of Laboratory Animals (Ministry of Science and Technology of China, 2016) and approved by the Animal Ethics Committee of Tianjin University of Science and Technology. No experiments involved human participants. The animals were kept in the SPF barrier system of the Institute of Radiation Medicine, Chinese Academy of Medical Sciences, with indoor temperatures of (23 ± 2°C), relative humidity (50 ± 10)%, and a 12 h light/dark cycle. The animals were given free access to food and water.

After one week of adaptive feeding, 50 healthy male SD rats were randomly divided into five groups according to their body weight. 10 rats served as the normal control group (CK, n = 10), and the remaining 40 were randomly divided into four groups, namely the high-fat model group (HFS, n = 10), the lovastatin group (LOV, n = 10), red yeast rice group (RYR, n = 10), and monascus pigment group (PIG, n = 10). This resulted in 10 animals in each group, who were all reared in separate cages. The rats in the CK group continued to be fed with basic feed and were given normal saline from 9:00 to 10:00 every morning. High-fat emulsions were force-fed to the HFS group [the emulsion was made up of lard (15%), cholesterol (2.5%), egg yolk powder (2.5%), glucose (5%), Tween-80 (2%), and distilled water (74%)]. Every day in the afternoon from 16:00 to 17:00, normal saline (HFS), lovastatin (LOV) 2 mg/kg·d, red yeast rice (RYR) 6 mg/kg·d (contains 3% lovastatin), and monascus pigment (PIG) 6 mg/kg·d were given through intragastric intervention for eight weeks. During the experiment, the food intake and water intake of each group of animals were determined regularly every day, and the body weight was determined once a week.

After eight weeks of feeding, rats in each group were fasted for 12 h, weighed, and had their body length measured before sacrifice. Rats were anesthetized with sodium pentobarbital at a dose of 30–50 mg/kg, blood samples were collected from the heart, anticoagulated, centrifuged at 3,500 rpm for 10 min, and stored at −80°C until measurement. The liver, heart, spleen, kidney, and perirenal fat pad were quickly separated, the organs rinsed with saline, blot dried with filter paper, and then the organs and tissues were weighed. The liver of each group of rats was washed with phosphate buffered saline (PBS), placed in a liquid nitrogen test tube, and then stored at −80°C until further use. Colon contents were then collected, quickly frozen in liquid nitrogen, and stored at −80°C until high-throughput sequencing.

### Measurements of Biochemical Parameters

The serum concentration of total cholesterol (TC), total triacylglycerol (TG), high-density lipoprotein cholesterol (HDL-C), low-density lipoprotein cholesterol (LDL-C), alanine aminotransferase (ALT), and aspartate aminotransferase (AST) were determined with the corresponding kit instructions (n = 8).

### Total RNA Extraction

Total RNA was extracted from the liver tissue using TRIzol reagent (Invitrogen Co., Carlsbad, CA, United States). RNA concentration and purity were estimated using the NanoDrop 2000 instrument (Thermo Scientific, United Kingdom). RNA integrity was assessed using the RNA Nano 6000 Assay Kit of the Agilent Bioanalyzer 2100 system (Agilent Technologies, CA, United States). Total RNA with good quality was used for further experiments.

### Library Construction and Sequencing

After total RNA was extracted, eukaryotic mRNA was enriched by Oligo (dT) beads, while prokaryotic mRNA was enriched by removing rRNA by Ribo-ZeroTM Magnetic Kit (Epicentre). Then the enriched mRNA was fragmented into short fragments using fragmentation buffer and reverse transcripted into cDNA with random primers. Second-strand cDNA were synthesized by DNA polymerase I, RNase H, dNTP, and buffer. Then the cDNA fragments were purified with the QiaQuick PCR extraction kit, end repaired, poly(A) added, and ligated to Illumina sequencing adapters. The ligation products were size selected by agarose gel electrophoresis, PCR amplified, and sequenced using Illumina HiSeqTM 2500 by Gene Denovo Biotechnology Co. (Guangzhou, China).

### Analysis of Sequencing Data

The raw reads obtained from the sequencing machines is processed as follows to obtain high quality clean reads: 1) removing reads containing adapters; 2) removing reads containing more than 10% of unknown nucleotides (N); and 3) removing low quality reads containing more than 50% of low quality (Q-value ≤ 20) bases.

Gene abundances were quantified by software RSEM (Li and Dewey, 2011). The gene expression level was normalized by using FPKM (Fragments Per Kilobase of transcript per Million mapped reads) method, and the formula is shown as follows:

$$FPKM=\frac{10^{6}C}{NL/10^{3}}$$

Principal component analysis (PCA) was performed with R package gmodels (http://www.r-project.org/) in this experiment.

### Differentially Expressed Genes and Enrichment Analysis

To identify differentially expressed genes across samples or groups, the edgeR package (http://www.rproject.org/) was used. We identified genes with a fold change ≥2 and a false discovery rate (FDR) < 0.05 in a comparison as significant DEGs. DEGs were then subjected to enrichment analysis of GO functions and KEGG pathways (n = 3).

### Real-Time Quantitative PCR Analysis

Total RNA was extracted from mycelia using the Plant RNA Kit (Omega). First-strand cDNA was synthesized using the PrimeScript 1st Strand cDNA Synthesis Kit (TaKaRa), with the Oligo dT Primer 15. Gene expression was monitored by RT-qPCR and carried out using the SYBR Premix Ex Taq II (TaKaRa). RT-qPCR was performed using the Stratagen Mx3000P (Agilent) with the following cycling program: hold at 95°C for 30 s, followed by a three-step PCR (42 cycles of denaturation at 95°C for 5 s, annealing at 60°C for 30 s, and extension at 72°C for 30 s) and dissociation curve analysis (at 95°C for 15 s, annealing at 60°C for 30 s, then collecting the dissociation curve from 60°C to 95°C, finally at 95°C for 15 s). The 2^−∆∆CT^ method was used to analyze the relative expression levels. Primer sequences used for quantitative PCR are shown in Table 1 (n = 3).

**TABLE 1:** The primer sequences of real-time PCR.

Gene	Primer sequence
CYP7A1	Forward primer: 5′-GGT​TGA​TTC​CGT​ACC​TGG​GC-3′
	Reverse primer: 5′-ACT​TTG​TGG​TAT​GAC​AGG​GAG​T-3′
LPIN	Forward primer: 5′-CGC​TCC​CGA​GAG​AAA​GTG​GT-3′
	Reverse primer: 5′-GGG​ATG​ACT​TCC​TGA​TCG​TTG​T-3′
CYP2C	Forward primer: 5′-TGA​AGG​ACA​TCC​GTC​AAT​CAA-3′
	Reverse primer: 5′-CAG​TAG​GCT​GTG​AGC​CGA​AA-3′
sPLA2	Forward primer: 5′-ATG​CCA​CAG​ATT​GGT​GCT​GT-3′
	Reverse primer: 5′-CCC​CCT​CGG​TAG​GAG​AAC​TT-3′

### GC/MS-Based Hepatic Metabolomics Analysis

Samples from the CK, HFS, LOV, RYR, and PIG groups were selected for untargeted metabolomics analysis (n = 3). Three biological replicates per group were considered. Samples were thawed at 4°C on ice. Then 100 μL of sample was taken and placed in an EP tube, then extracted with 300 μL of methanol, added to 10 μL internal standard substances, and vortexed for 30 s, then treated with ultrasound for 10 min (incubated in ice water) and incubated for 1 h at −20°C to precipitate proteins. Then the sample was centrifuged at 12,000 rpm for 20 min at 4°C. The supernatant (200 μL) was then transferred into a fresh 2 ml LC/MS glass vial and 10 μL from each sample was taken and pooled as QC samples. 150 μL supernatant was taken for the UHPLC-QTOF-MS analysis.

LC-MS/MS analyses were performed using an UHPLC system (1290, Agilent Technologies) with a UPLC BEH Amide column (1.7 μm 2.1 × 100 mm, Waters) coupled to TripleTOF 5600 (Q-TOF, AB Sciex). The Triple TOF mass spectrometer was used for its ability to acquire MS/MS spectra on an information-dependent basis (IDA) during an LC/MS experiment. MS raw data (.raw) files were converted to the mzML format using ProteoWizard and processed by R package XCMS (version 3.2), including retention time alignment, peak detection, and peak matching. R package CAMERA was used for peak annotation after XCMS data processing. In-house MS2 database was applied in metabolites identification.

### DNA Extraction and 16S rRNA Gene Sequencing

Microbial DNA was extracted from stool samples using the E.Z.N.A. stool DNA Kit (Omega Biotek, Norcross, GA, United States) according to manufacturer’s protocols (n = 3). The 16S rDNA V3-V4 region of the Eukaryotic ribosomal RNA gene were amplified by PCR (95°C for 2 min, followed by 27 cycles at 98°C for 10 s, 62°C for 30 s, 68°C for 30 s, and a final extension at 68°C for 10 min) using primers 341F: CCTACGGGNGGCWGCAG; 806R: GGACTACHVGGGTA TCTAA T, where the barcode is an eight-base sequence unique to each sample. PCR reactions were performed in triplicate 50 μL mixtures containing 5 μL of 10× KOD Buffer, 5 μL of 2.5 mM dNTPs, 1.5 μL of each primer (5 μM), 1 μL of KOD Polymerase, and 100 ng of template DNA.

Amplicons were extracted from 2% agarose gels and purified using the AxyPrep DNA Gel Extraction Kit (Axygen Biosciences, Union City, CA, United States) according to the manufacturer’s instructions and quantified using QuantiFluor-ST (Promega, United States). Purified amplicons were pooled in equimolar and paired-end sequenced (2 × 250) on an Illumina Hiseq2500 sequencing platform according to the standard protocols. The raw reads were deposited into the NCBI Sequence Read Archive (SRA) database (Accession Number: PRJNA670295).

### Pearson Correlation Coefficient Model

Pearson correlation coefficients were calculated for metabolome and transcriptome data integration. Gene and metabolite pairs were ranked in the descending order of absolute correlation coefficients. The top 50 genes and metabolites were selected for heatmap analysis using pheatmap packages in R project (Kolde, 2015). Additionally, the top 250 pairs of genes and metabolites (with the absolute Pearson correlation > 0.5) were applied for metabolitetranscript network analysis using igraph packages in R project (Csardi, 2006). The Pearson correlation coefficient between every level of microbiota and metabolomic datasets were calculated in R (version 3.5.1). The correlation heatmap was generated using pheatmap package in R.

### Statistical Analysis

All data were expressed as the mean ± standard error of the mean (SEM). Statistical group analysis was performed by one-way analysis of variance (ANOV A) followed by Dunnett’s multiple comparison test using Graphpad Prism 7.0 software (GraphPad software, San Diego, CA, United States). *p < 0.05 and #*p* < 0.05 were set as statistical significance.

## Results and Discussion

### Effects of Red Yeast Rice and Monascus Pigment on Body Weight and Organ Quality of Rats

**FIGURE 1:**
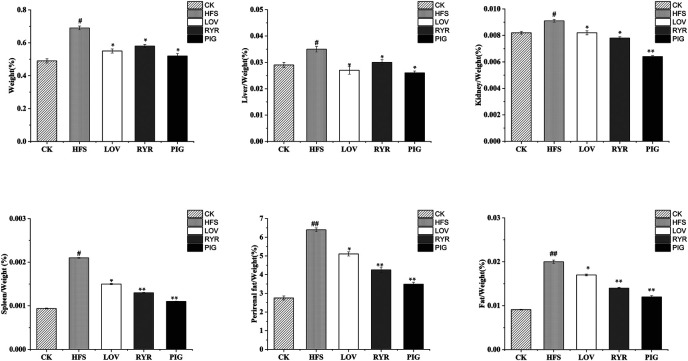
Determination results of body mass and viscera quality of different groups of rats (n = 8). Note: Compared with CK group, “#” means significant difference (*p* < 0.05), “##” means extremely significant difference (*p* < 0.01); Compared with HFS group, “*” showed significant difference (*p* < 0.05), and “**” showed extremely significant difference (*p* < 0.01), the same as below.

The results of rat body weight and organ quality of each group are shown in Figure 1. Compared with HFS, after the intervention of LOV, RYR, and PIG, the weight growth rate of rats decreased by 20.29, 15.94, and 24.64% respectively (*p* ≤ 0.05)., Liver/Weight (%) was decreased by 22.86, 14.29, and 25.71%, respectively (*p* ≤ 0.05), Kidney/Weight (%) decreased by 9.89% (*p* ≤ 0.05), 14.29% (*p* ≤ 0.05), and 29.67% (*p* ≤ 0.01), Spleen/Weight (%) decreased by 28.57% (*p* ≤ 0.05), 38.09% (*p* ≤ 0.01), and 47.62% (*p* ≤ 0.01)), and Perirenal fat/Weight (%) reduced by 20.18% (*p* ≤ 0.05), 33.53% (*p* ≤ 0.01), and 45.48% (*p* ≤ 0.01). There was no significant difference between the LOV group and RYR group (*p* > 0.05) in the changes of SD rats’ body mass and all organ indexes. The effect of PIG on reducing body weight and blood lipid was better than that of RYR and LOV, indicating that PIG may have a unique mechanism in controlling lipid metabolism.

### Effects of Red Yeast Rice and Monascus Pigment on Blood Lipid and Liver Function in Rats

**FIGURE 2:**
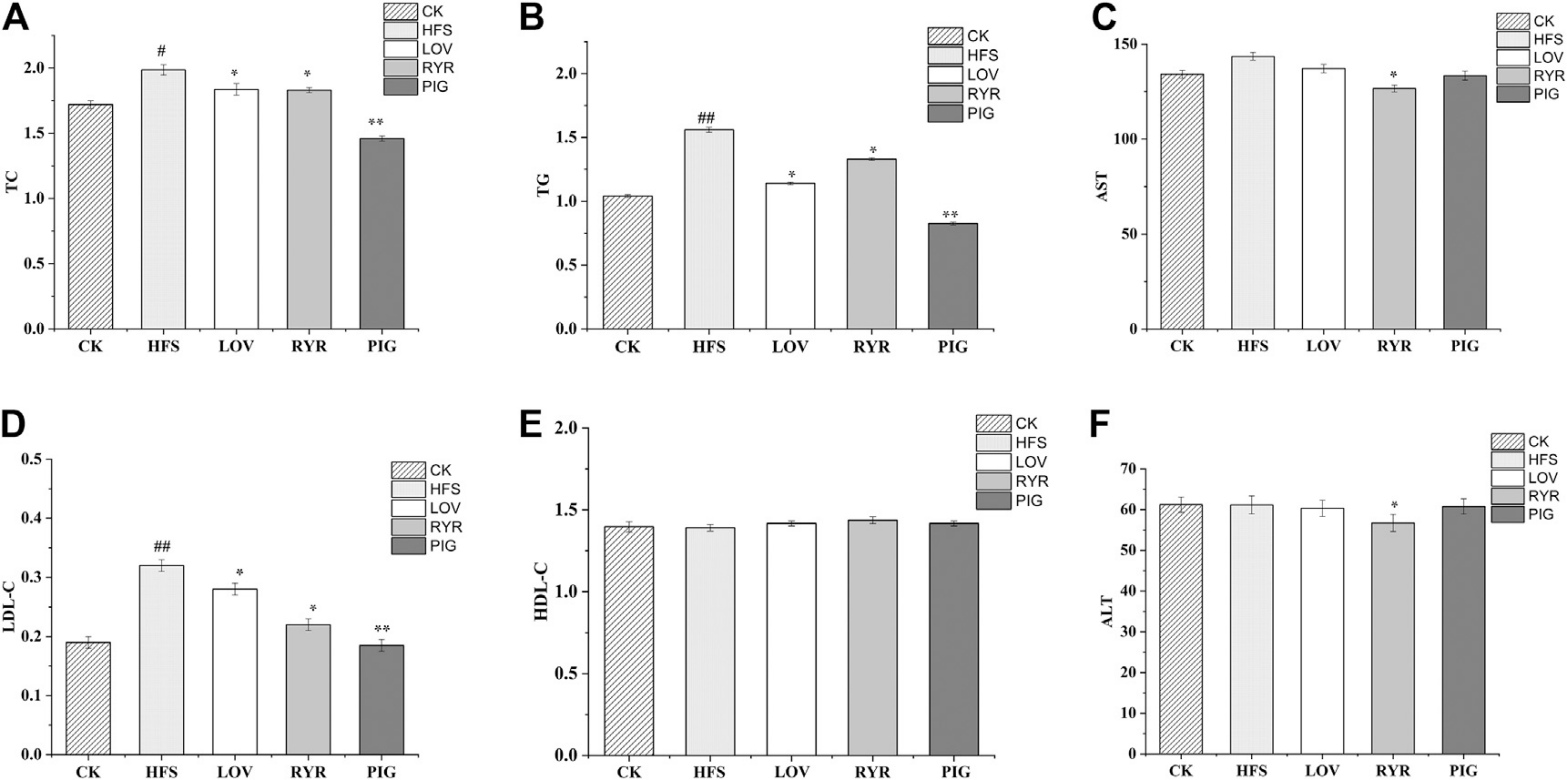
Determination results of blood lipid indexes of different groups of rats (n = 8).

As shown in Figure 2, compared with HFS, after the intervention of LOV, RYR, and PIG, TC of rats in each group was reduced by 11.54% (*p* ≤ 0.05), 12.02% (*p* ≤ 0.05), and 30.8% (*p* ≤ 0.01), respectively; TG was reduced by 26.92% (*p* ≤ 0.05), 14.1% (*p* ≤ 0.05), and 47.44% (*p* ≤ 0.01), respectively; LDL-C decreased by 12.5% (*p* ≤ 0.05), 31.25% (*p* ≤ 0.01), and 42.19% (*p* ≤ 0.01), respectively; HDL-C did not change significantly (*p* > 0.05). There was no significant difference in blood lipid indexes between the LOV group and the RYR group (*p* > 0.05); this may be because the main lipid-lowering component in RYR is also Monacolin K. However, compared with LOV and RYR, the PIG group had lower levels of TC, TG, and LDL-C (*p* ≤ 0.05).

The liver function of rats was damaged by the long-term high glucose and high-fat diet. Compared with CK, the HFS group AST increased by 6.83%, however the ALT level had no significant change (*p* > 0.05). LOV and PIG did not change the level of ALT and AST significantly (*p* > 0.05), while the levels of ALT and AST in the RYR group decreased by 7.27 and 11.49%, respectively. Elevated levels of AST and ALT is related to abnormal lipid metabolism and inflammatory damage (Yang et al., 2018). The result indicated that RYR may have a protective effect on liver damage caused by a high-fat diet.

### Principal Component Analysis

**FIGURE 3:**
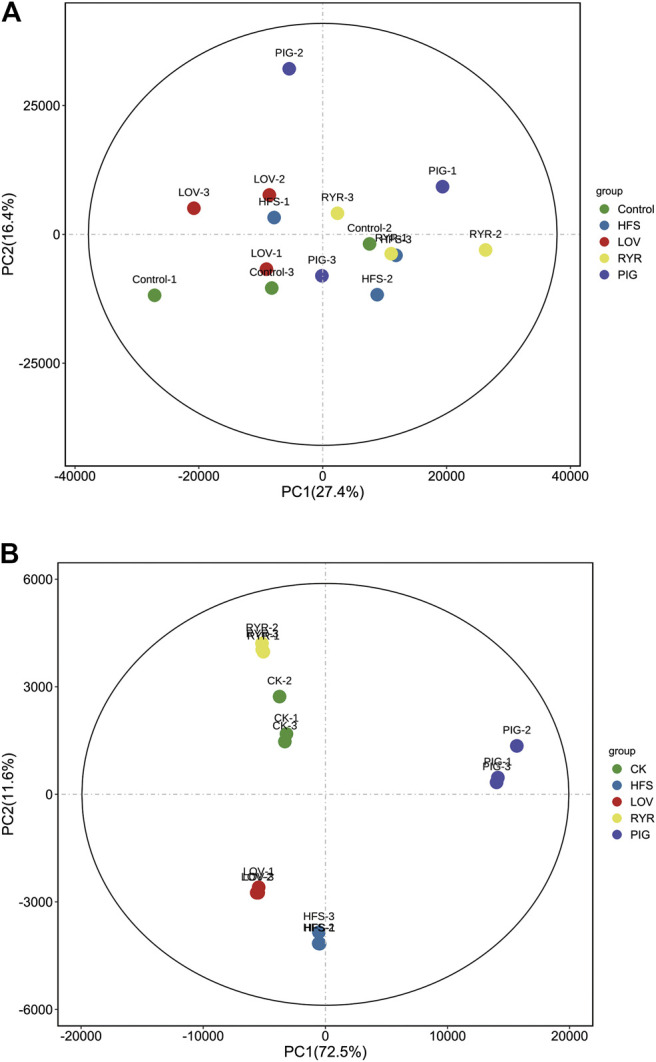
Metabolic **(A)** and Gene **(B)** profile of the CK, HFS, LOV, RYR, and PIG groups visualized by principal component analysis (PCA) (n = 3).

Principal component (PCA) analysis over the transcriptome and metabonomic profiles of the fifteen samples was performed respectively (three biological replicates per group), which showed the variance within the datasets. Figure 3A is the principal component analysis diagram of the metabolome. There is no significant difference in the positive and negative ion mode. Figure 3B is the principal component analysis diagram of the transcriptome, and there is no significant difference in gene expression in each group.

### Joint Analysis of Liver Transcriptome and Liver Lipid Metabolome in SD Rats

**FIGURE 4:**
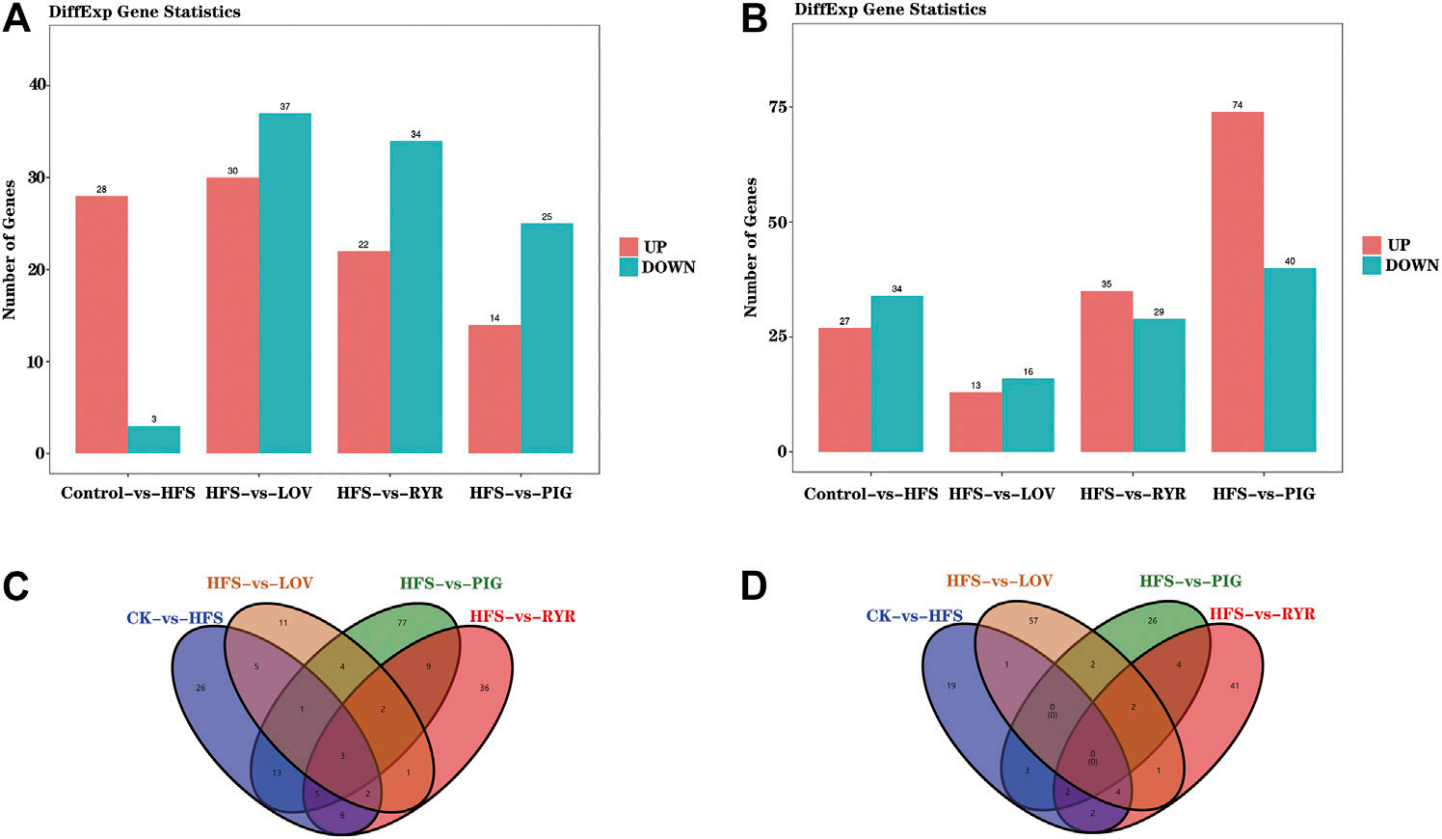
**A)** Statistical histogram of differential genes between samples; **(B)** Statistical histogram of differential metabolites (In **(A)** and **(B)**, the x-coordinate is samples compared in pairs, and the vertical table is the number of differentially expressed genes or metabolites; Green and red represent differentially expressed genes or metabolites that are down-regulated and up-regulated, respectively); **(C)** Venn diagram showing the differentially expressed genes shared by the mice from each group; **(D)** Venn diagram showing the differentially expressed metabolites shared by the mice from each group (n = 3).

The effects of LOV, RYR, and PIG on liver metabolites in high-fat model rats through liver lipid metabolomics combined with liver transcriptomics and the metabolic pathways was explored. FDR and log2FC were used to screen differential genes and metabolites (FDR < 0.05 and |log2FC|>1), and the results are shown in Figure 4.

Compared with the CK group, HFS, LOV, RYR, and PIG had 61, 29, 64, and 114 different genes respectively, among which there are 27, 13, 35, and 74 up-regulated genes and 34, 16, 29, and 40 down-regulated genes. In terms of metabolites, HFS, LOV, RYR, and PIG have 31, 67, 56, and 39 differential metabolites respectively, of which 28, 30, 22, and 14 are up-regulated and 3, 37, 34, and 25 are down-regulated (Figures 4A,B). The Venn diagrams of differential genes and differential metabolites between groups are shown in Figures 4C,D.

**FIGURE 5:**
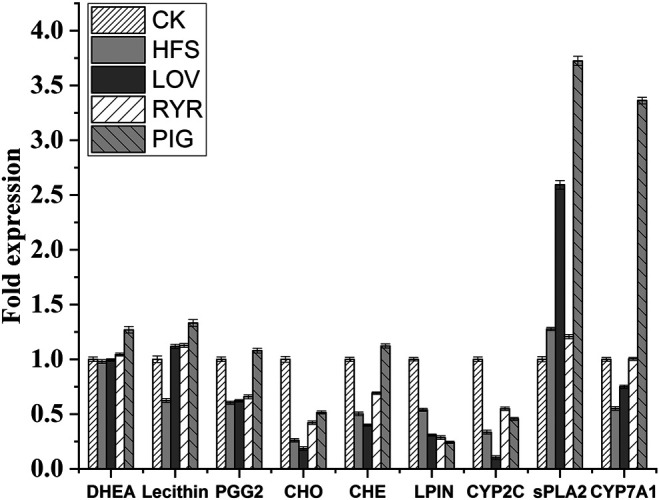
Changes in metabolites and genes in metabolic pathways (The red circles indicate the metabolites with up-regulated abundance, the green circles indicate the metabolites with down-regulated abundance, the red boxes indicate genes whose expression levels are up-regulated, and the green boxes indicate genes whose expression levels are down-regulated).

Compared with HFS, the LOV, RYR, and PIG groups have the same gene and metabolite changes in the three metabolic pathways of Steroid hormone biosynthesis, Glycerolipid metabolism, and Arachidonic acid metabolism pathway, as shown in A, B, and C in Figure 5.

In the metabolic pathway of Steroid hormone biosynthesis (Figure 5A), the level of metabolite Dehydroepiandrosterone (DHEA) increases, and cholesterol 7alpha-monooxygenase (CYP7A1) is up-regulated. Cholesterol can be broken down into Pregnenolone by cholesterol monooxygenase (side-chain-cleaving) (CYP11A), and then further broken down into DHEA by 17alpha-hydroxyprogesterone deacetylase (CYP17A). DHEA can be further decomposed into 7alpha-Hydroxydehydroepiandrosterone by CYP7A1. Therefore, the increase in DHEA content means that more cholesterol is broken down in this pathway.

In the Glycerolipid metabolism pathway (Figure 5B), the level of phosphatidate phosphatase LPIN (LPIN) is down-regulated. Glycerol can be broken down into Phosphatidate by a series of enzymes. LPIN can hydrolyze Phosphatidate into 1,2-Diacyl-sn-glycerol (DAG). DAG can further synthesize Triacylglycerol. LPIN was down-regulated to inhibit the conversion of phosphatidylcholine to DAG. Therefore, in the Glycerolipid metabolism pathway, LOV, RYR, and PIG can reduce the synthesis of triglycerides by inhibiting the expression of LPIN.

In the Arachidonic acid metabolism pathway (Figure 5C), the level of metabolites Lecthin and ProstaglandinG2 (PGG2) increases, and Secretory phospholipaseA2 (sPLA2) and CYP2C (cytochrome P450, family 2, subfamily C) also increased. Lecithin is decomposed into Linoleic Acid (LA) under the action of sPLA2, and then it is further hydrolyzed into Arachidonic acid (AA) (Bannehr et al., 2019). Under the action of CYP2C, arachidonic acid is metabolized to produce prostaglandinG2 (PGG2), and then prostaglandinH2 (PGH2). PGH2 relies on the catalytic action of COX-2 in the blood vessel wall to produce prostaglandinI2 (PGI2). PGI2 is considered to play a role in the cardiovascular system health, especially through smooth muscle relaxation and inhibition of platelet aggregation, which has a strong vasodilator effect (Lee et al., 2016). Compared with LOV, RYR has a differential expression of genes and metabolites in the Circadian entrainment and Estrogen signaling pathway; this may be due to the metabolic changes caused by other non-statin substances in RYR. In the Circadian entrainment pathway, the circadian proteins, such as period circadian protein 2 (PER2), is the main regulator in the biosynthesis of lipids. The function of circadian clock genes is related to metabolic phenotype and lipid absorption. The circadian clock plays its role by regulating the key steps of lipid metabolism (Julius et al., 2019). In the Estrogen signaling pathway, estrogen and its receptors can affect adipocyte differentiation, promote lipolysis, and inhibit lipid synthesis, thereby reducing fat deposition in adipose tissue (Watanabe et al., 2004).

**FIGURE 6:**
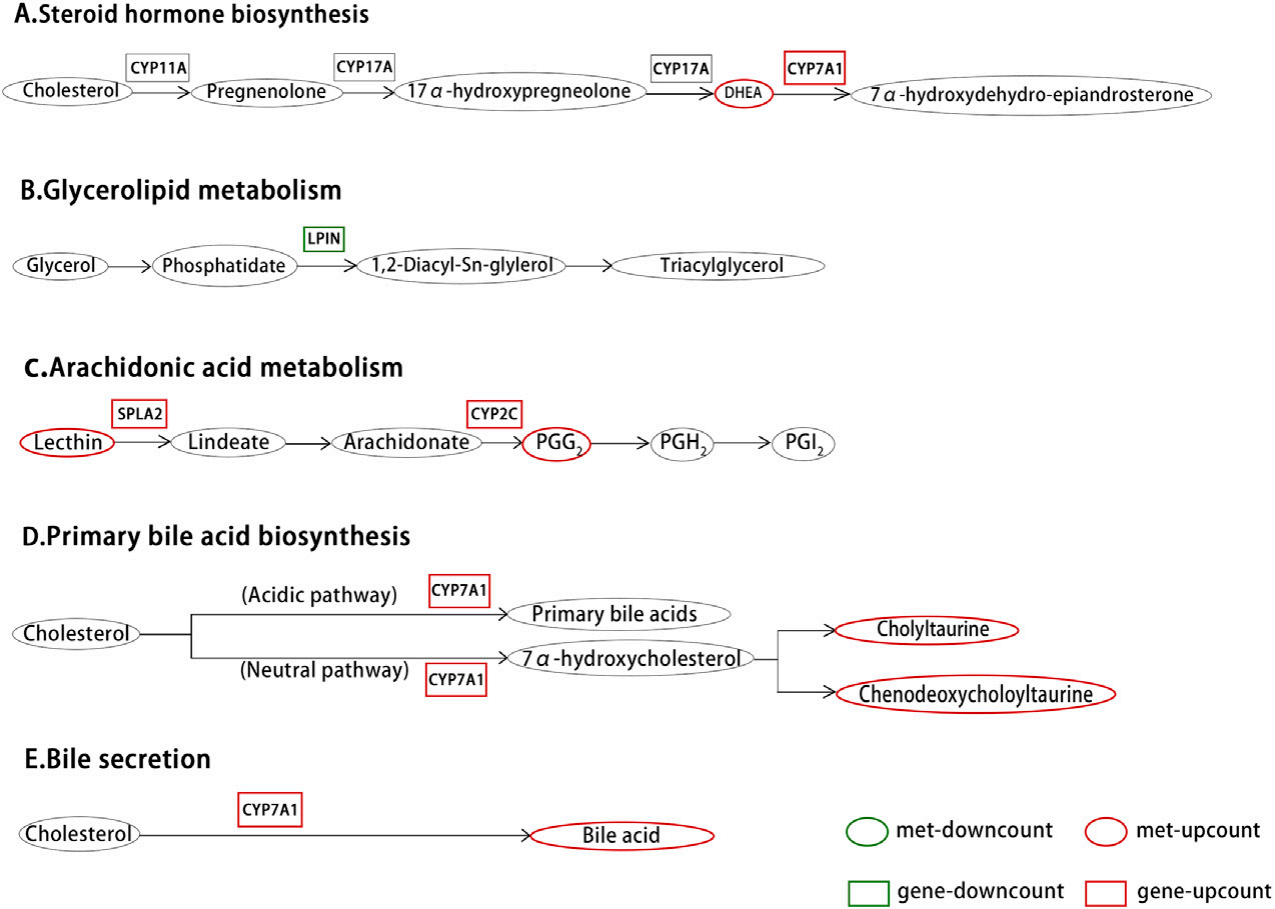
Differential metabolites and differential gene expression statistics histogram (n = 3).

Compared with HFS, LOV, and RYR, the PIG group has significant differences in the genes and metabolites of the two pathways, Primary bile acid biosynthesis and Bile secretion pathway. In the Primary bile acid biosynthesis pathway (Figure 5D), cholesterol metabolism has two pathways. In the Acidic pathway, cholesterol is metabolized to 7alpha, 27-Dihydroxycholesterol through CYP7A1, and then further metabolized to primary bile acid. In the neutral pathway, cholesterol is metabolized by CYP7A1 to 7alpha-Hydroxycholesterol, and then further catalyzed by HSD3B7 to 7alpha-Hydroxycholest-4-en-3-one. Then one part is catalyzed into Chenodeoxycholoyltaurine (CHE) by a series of enzymes, and the other part is catalyzed into Cholyltaurine (CHO). Similarly, in the Bile secretion pathway (Figure 5E), CYP7A1 was also observed to promote bile secretion by metabolizing cholesterol to bile acid. CYP7A1 is the rate-limiting enzyme in the liver bile acid biosynthesis pathway (Chambers et al., 2019). It promotes the secretion of biliary cholesterol by converting excess cholesterol into bile acids, and plays an important role in regulating lipid, glucose, and energy metabolism and liver cholesterol homeostasis (Ferrell et al., 2016; Liu et al., 2016). Studies have shown that CYP7A1 deficiency in the human body is related to hypercholesterolemia and premature atherosclerosis (Pullinger et al., 2002). Therefore, through the up-regulation of CYP7A1 in the two pathways - bile acid biosynthesis and bile secretion and metabolism - pigment showed a more powerful cholesterol lowering effect. Pan Ziming et al. found that monascin and Ankaflavin reduced oleic-acid-induced steatosis in mouse FL83B hepatocytes by inhibiting triglyceride production and promoting fatty acid -oxidation. Interestingly, although the PIG we used in this study also contains a small amount of Anka pigment, it was not found that the PIG group has the same metabolic regulation pathway as Anka. The expression and change trends of specific metabolites and differential genes between different groups are shown in Figure 6.

### Effects of Administration on Lipid Metabolism Related Gene Expression

**FIGURE 7:**
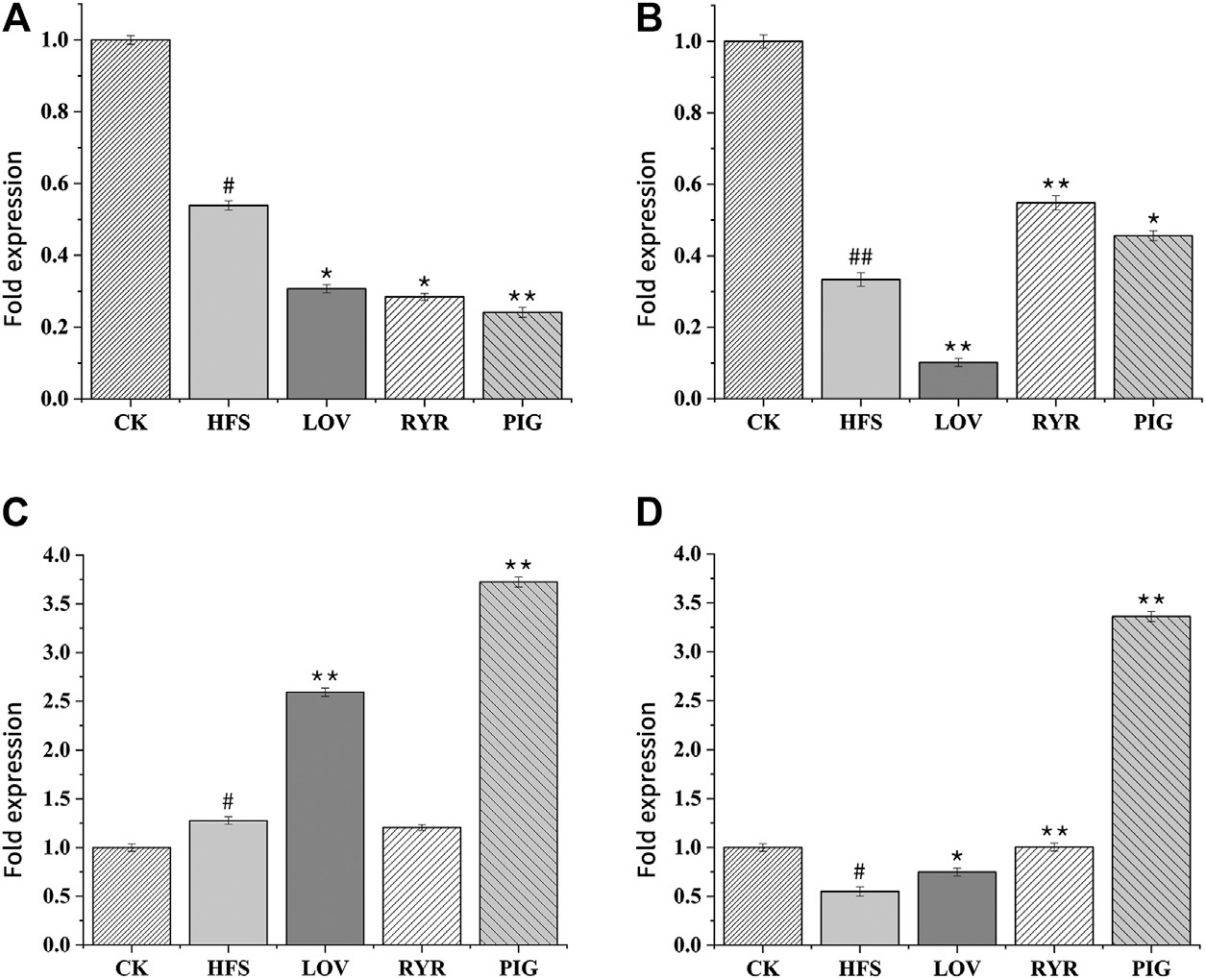
Effects of HFS, LOV, RYR, and PIG on mRNA expression in liver-related genes of hyperlipidemic rats (n = 3). **(A)** phosphatidate phosphatase LPIN; **(B)** CYP2C; **(C)** sPLA2; **(D)** CYP7A1. The mRNA levels of the CK group were used as the reference value. Data were expressed as the relative mRNA level for each gene and represented as the mean ± SD (n = 3).

The RT-PCR results are shown in Figures 7A–D are the expressions of LPIN, CYP2C, sPLA2, and CYP7A in the rat liver before and after administration, respectively. Compared with the CK group, the expression levels of LPIN, CYP2C, and CYP7A1 mRNA in the liver tissue of the HFS group were significantly decreased, and the expression level of sPLA2 mRNA was significantly increased. Compared with the HFS group, the relative expression of LPIN and CYP2C gene mRNA in the liver of the LOV group was significantly reduced, and the relative expression of sPLA2 and CYP7A1 gene mRNA was significantly increased. The relative expression of LPIN gene mRNA in the liver of the RYR group significantly decreased, and the relative expression of CYP2C and CYP7A1 gene mRNA was extremely significantly increased. The relative expression of LPIN gene mRNA in the liver of the PIG group significantly decreased, and the relative expression of sPLA2, CYP2C, and CYP7A1 gene mRNA was extremely significantly increased. In summary, LOV, RYR, and PIG may reduce blood lipids by promoting the expression of sPLA2, CYP2C, and CYP7A1 genes in rat livers, inhibiting the expression of the LPIN gene, thereby inhibiting the production of cholesterol in the body and promoting the decomposition of cholesterol.

### Effect of Drug Intervention on Intestinal Flora of High-Fat Rats

**FIGURE 8:**
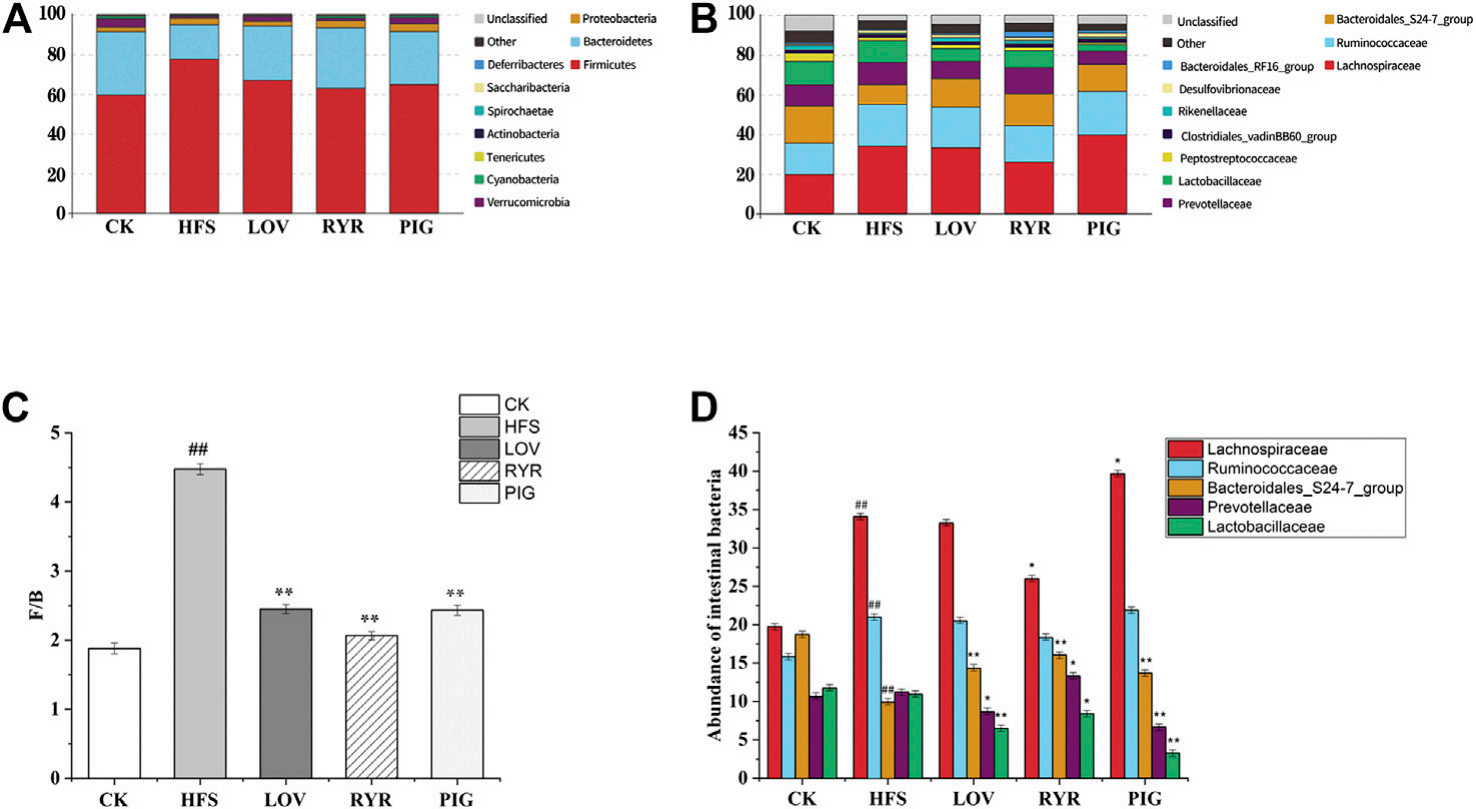
Composition of the gut microbiota (n = 3). **(A)** Gut microbiota composition at the phylum level. **(B)** Gut microbiota composition at the family level. **(C)** F/B ratio (Firmicutes/Bacteroides). **(D)** Community abundance on phylum level.

A total of 10 bacterial phyla were detected in the intestinal flora of rats in each group, mainly composed of *Firmicutes*, *Bacteroidetes*, *Proteobacteria*, and *Verrucomicrobia*, as shown in Figure 8A. The sum of the relative abundance of *Firmicutes* and *Bacteroidetes* is greater than 90%, which is the main dominant phylum in the intestinal contents of rats (Vacca et al., 2020). The changes in the composition of intestinal flora in SD rats after intervention of a high-fat diet may be one of the causes of obesity and hyperlipidemia in rats (Zeng et al., 2016; Liu et al., 2017; Kiran et al., 2019). Compared with the CK group, the relative abundance of Firmicutes in the intestinal contents of rats in the HFS group increased by 30.16%, and the relative abundances of *Bacteroidetes* and *Verrucomicrobia* decreased by 45.33 and 80.97%, respectively.

Compared with HFS, the relative abundance of *Firmicutes* in the LOV, RYR, and PIG groups decreased by 13.74, 18.91, and 16.51%; *Bacteroidetes* increased by 57.53, 75.69, and 53.55%, respectively. The relative abundance of *Verrucomicrobia* increased by 63.94, 37.03, and 75.11%, respectively. The ratios of *Firmicutes*/*Bacteroidetes* were all significantly reduced (*p* ≤ 0.01), but there was no significant difference between the three groups of LOV, RYR, and PIG (*p* > 0.05), as shown in Figure 8C. *Bacteroides* and *Firmicutes* are the main components of the intestinal flora, and their proportions change as diet and age change (Koliada et al., 2017). Degradation of dietary polysaccharides and fiber by *Bacteroides* and *Firmicutes* in the gut results in the production of SCFAs, such as propionate, acetate, and butyrate. Propionate is an important energy source for the host via *de novo* synthesis of lipids and glucose in the liver (Shabana et al., 2018). *Verrucomicrobia* has anti-inflammatory, anti-atherosclerotic, and insulin sensitivity effects (Schneeberger et al., 2015). The increased relative abundance of *Verrucomicrobia* can cause changes that are beneficial to metabolism and help mice reduce obesity (Fjære et al., 2019).

Compared with CK, the relative abundance of *Lachnospiraceae* and *Ruminococcaceae* in the HFS group increased by 72.60% (*p* ≤ 0.01) and 32.72% (*p* ≤ 0.01); the relative abundance of *Bacterooidales-S24-7*-group decreased by 47.07% (*p* ≤ 0.01); *Prevotellaceae* had no significant change (Figure 8B). A high-fat diet can increase the relative abundance of lachnospiraceae and then disrupt glucose metabolism, causing inflammation and promoting the onset of T1D (Kostic et al., 2016; Zeng et al., 2016). The different OTUs of *Lachnospiraceae* are related to changes in lipid metabolism, as well as specific nutrients such as saturated fat and total fat, and thus to obesity (De Flippis et al., 2015). High-fat diet, antibiotic use, and sweet food intake will increase the abundance of *Ruminococcaceae* (Shang et al., 2016; Owen et al., 2017). Excessive *Ruminococcaceae* may damage the intestinal mucosa, which is not conducive to intestinal health. *Bacterooidales-S24-7*-group is a novel branch of the “*Bacteroides* group” first recognized in 2002 (Salzman et al., 2002; Ormerod et al., 2016).

Compared with HFS, after the intervention of LOV, RYR, and PIG, the abundance of *Bacteroidales-S24-7*-group in the rat intestine increased by 44.49, 61.85, and 37.94%, respectively; the difference was extremely significant (*p* ≤ 0.01). The abundance of *Lactobacillaceae* decreased by 40.63% (*p* ≤ 0.01), 23.12% (*p* ≤ 0.05), and 69.91% (*p* ≤ 0.01), respectively. *Bacteroides* is one of the most abundant genera in the mammalian colon (Cavalcanti Neto et al., 2018). *Bacteroides* can actively refine the gut environment to make it more hospitable to themselves and other microorganisms (Olli et al., 2016). *Bacterooidales-S24-7*-group increasing can reduce the obesity induced by a high-fat diet (Gauffin Cano et al., 2012), which may be an important reason for PIG, LOV, and RYR to reduce high-fat diet induced obesity.

Compared with HFS, the abundance of *Lachnospiraceae* in the LOV group and the RYR group decreased by 2.51 and 23.78%, respectively (*p* ≤ 0.05). The abundance of *Lachnospiraceae* is positively correlated with glucose and/or lipid metabolism, and induces metabolic disorders (Salonen et al., 2014; Lippert et al., 2017; Chávez-Carbajal et al., 2019). It is worth noting that the abundance of *Lachnospiraceae* in the PIG group increased by 16.35% (*p* ≤ 0.05); this indicates that *Lachnospiraceae* may also have some potential beneficial bacteria, such as *Roseburia* species, which is often associated with a healthy state and is one of the main SCFA producers (Park et al., 2011; Murugesan et al., 2015; Koh et al., 2016; Sun et al., 2016; La Rosa et al., 2019). *Roseburia* is the genus most involved in controlling intestinal inflammation, atherosclerosis, and immune system maturation (Atarashi et al., 2010; Kasahara et al., 2018). The abundance of *Prevotellaceae* which was related to high total carbohydrate and monosaccharide intake in the LOV group and PIG group decreased by 23.49% (*p* ≤ 0.05) and 40.80% (*p* ≤ 0.01), respectively, while the RYR group increased by 18.37% (*p* ≤ 0.05). *Prevotellaceae* can secrete polysaccharide hydrolase and decompose non-fibrous polysaccharides in the intestine as an energy source. The increase of *Prevotellaceae* can promote the development of obesity (Wu et al., 2011; White et al., 2014). In addition, *Prevotellaceae* has been widely associated with a high dietary fiber intake (Gual-Grau et al., 2019). Therefore, changes are different in the abundance of *Prevotellaceae* in the LOV, PIG, and RYR groups and this may be due to there being almost no dietary fiber in the LOV and PIG groups and the large amounts of dietary fiber in RYR.

### Correlation Analysis of Metabolomics and Microbiomics Data

**FIGURE 9:**
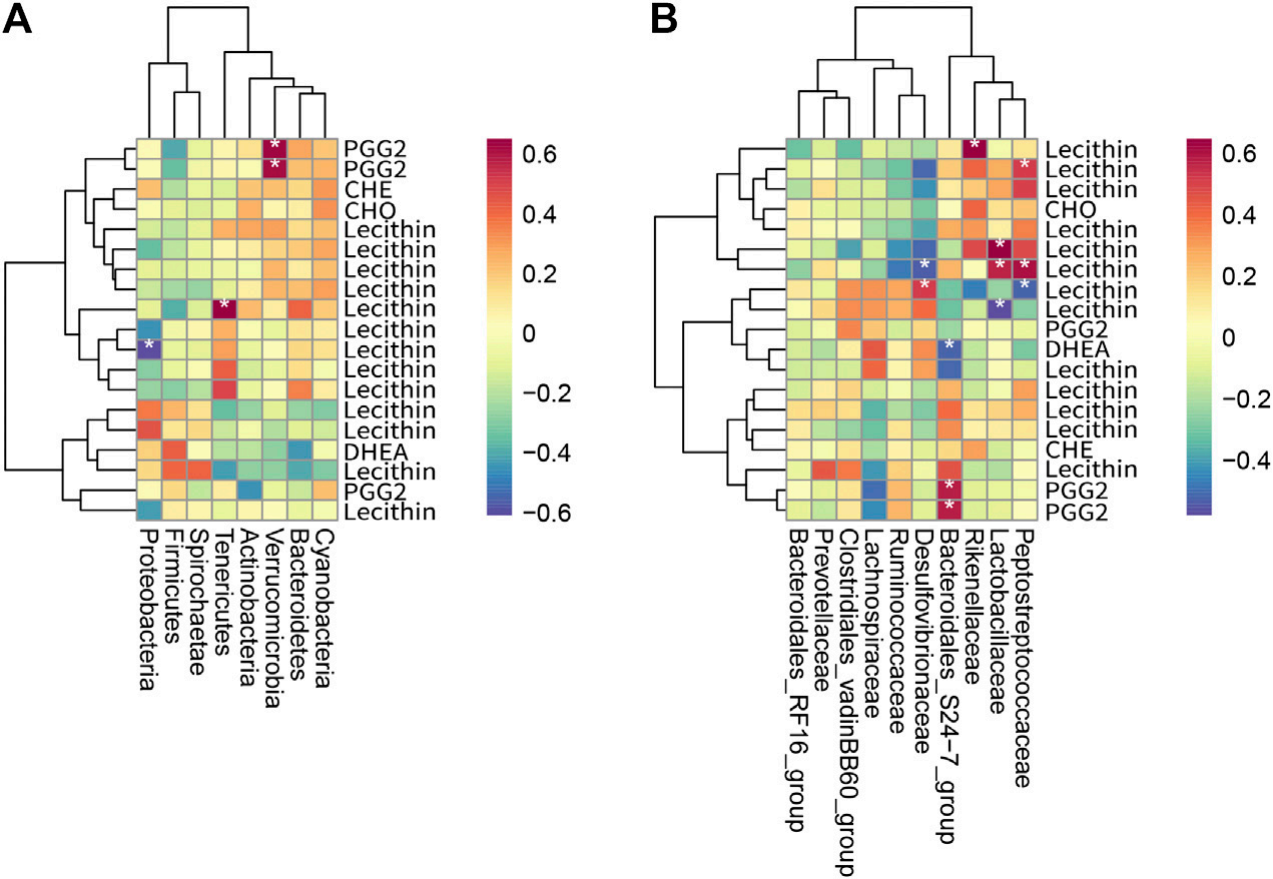
Heat map of the correlation between metabolites and intestinal bacteria (n = 3). **(A)** shows a heat map of intestinal bacteria and differential metabolites at the phylum level, and **(B)** shows a heat map of intestinal bacteria and differential metabolites at the family level. The horizontal axis is intestinal bacteria, and the vertical axis is metabolites. Each grid represents the correlation coefficient between intestinal bacteria and metabolites. The color changes from white to red, indicating the positive correlation is from weak to strong; from white to blue indicates the negative correlation is from weak to strong. “*” means significant correlation (*p* < 0.05); “**” means extremely significant correlation (*p* < 0.01).

The gut microbiota is a massive “organ” able to perform complex functions and thereby produce a myriad of differentially abundant metabolites. To investigate the functional correlation between the gut microbiome changes and host metabolome alterations, the R language pheatmap package (https://CRAN.R-project.org/package=pheatmap) was used to draw a heat map of the correlation between microorganisms and intestinal bacteria (as shown in Figure 9). Recent studies have shown that the imbalance of intestinal flora may be related to the occurrence and development of atherosclerosis, diabetes mellitus, and hyperlipidemia, and that microbial metabolites also play a protective or aggravating role in cardiovascular diseases (Tang et al., 2017; Zhu et al., 2020). Through studying the relationship between intestinal bacteria and metabolites, it is found that at the phylum level (Figure 9A), the abundance of *Verrucomicrobia* was significantly positively correlated with prostaglandin 2 (PGG2), which is involved in the metabolism of arachidonic acid. The abundance of *Proteobacteria* was significantly negatively correlated with Lecithin involved in the arachidonic acid metabolic pathway. At the family level (Figure 9B), the abundance of *Bacteroidales-S24-7-group* was significantly positively correlated with prostaglandin 2 (PGG2) involved in the arachidonic acid metabolic pathway. It has a significant negative correlation with dehydroepiandrosterone (DHEA), which is involved in the biosynthesis and metabolism of steroid hormones. The abundance of *Lactobacillaceae* is significantly positively correlated with Lecithin, which is involved in the metabolic pathway of arachidonic acid. Pfister and Campbell (Pfister and Campbell, 1996) have shown that diet-induced hypercholesterolemia can cause changes in arachidonic acid metabolism, but the specific mechanism of action has not been elucidated. Han et al. (Han et al., 2019) found that *Lactobacillus* intestinalis was highly correlated with testosterone, beta-estradiol, and prostaglandin E2, which were enriched in steroid hormone biosynthesis and arachidonic acid metabolism, by studying the antihypertensive mechanism of astragalus and salvia. Jones (Jones, 2004) found that arachidonic acid metabolites play a role in intestinal inflammation in diarrheal diseases. Ferrer and Moreno (Ferrer and Moreno, 2010) have shown that arachidonic acid and its derived eicosanoids play an important role in the intestinal epithelium. By converting arachidonic acid into PGH2, prostaglandins involved in the inflammatory process play an important role in the recovery of the intestinal barrier function of the ischemic pig ileum. For infants with necrotizing enterocolitis, increasing the intake of LCPUFA, including arachidonic acid, can promote the recovery of damaged intestinal tissue. Therefore, RYR, LOV, and PIG may regulate host lipid metabolism by regulating the interaction of intestinal bacteria with arachidonic acid and its metabolites.

## Conclusion

RYR is a common medicinal and edible food, and PIG is a common edible coloring and food additive. This study evaluated the effects of the two on blood lipids and weight loss in rats on a high-sugar and high-fat (HFS) diet. At the same time, the lipid-lowering mechanism of the two was studied through intestinal flora analysis, liver transcriptome analysis, and liver lipidome analysis. RYR and PIG can reduce fat accumulation and reduce blood lipid in rats induced by a HFS diet, and PIG is more effective than LOV and RYR in reducing body fat and cholesterol. Intestinal flora analysis showed that, after the intervention of LOV, RYR, and PIG in rats, there were significant differences in the phyla and family of intestinal bacteria. The intestinal environment was improved by changing the abundance and proportion of intestinal flora in rats. RYR contains lovastatin, which is still the main lipid-lowering substance. Therefore, RYR and LOV have the same metabolites and gene expression in Steroid hormone biosynthesis, Glycerophospholipid metabolism, and the Linoleic acid metabolism pathway. However, due to RYR containing a small amount of fragments, polysaccharides, and other substances in addition to lovastatin, there are differences in the diurnal entrained metabolic pathway and estrogen signaling pathway between RYR and LOV. This may be why RYR was more secure than LOV. PIG has lipid metabolism regulation pathways—bile acid biosynthesis and bile secretion and metabolism—which are not found in RYR and LOV, which may be the reason why PIG has a better cholesterol lowering effect.

Anka was considered to be the main lipid-lowering component in PIG. However, ir is difficult to use Anka in the food industry because of its low yield and difficulty to separate. PIG, as a mixture, is still widely used in the food industry. Compared with RYR and LOV, PIG has unique metabolic pathways, which may be the reason why PIG is superior to lovastatin and RYR in lowering blood lipids and promoting cholesterol breakdown. Monascus purple SHM1105 will have application potential in the development of a new type of cholesterol-lowering functional food. The results of this study prove that RYR and PIG have the effects of preventing obesity and assisting in lowering blood lipids, and have potential application value.

## Data Availability Statement

The datasets presented in this study can be found in online repositories. The names of the repository/repositories and accession number(s) can be found in the article/Supplementary Material.

## Ethics Statement

All animal procedures were performed in accordance with the Guidelines for Care and Use of Laboratory Animals (Ministry of Science and Technology of China, 2016) and approved by the Animal Ethics Committee of Tianjin University of Science and Technology.

## Author Contributions

Conceptualization, HY and CW; Performing the experiments, JW and LZ; Data processing, ZL, QG and RP; Writing and approving the manuscript, HY and JW. All authors contributed to the article and approved the submitted version.

## Funding

This research was funded by the National Natural Science Foundation of China (NSFC) under Grant number 31330059 and 31801519.

## Conflict of Interest

Author RP was employed by Zhejiang Sanhe Bio-Tech Co., Ltd. Zhejiang Sanhe Bio-Tech Co., Ltd. provided technical support for this study, such as strain and lovastatin detection.

The remaining authors declare that the research was conducted in the absence of any commercial or financial relationships that could be construed as a potential conflict of interest.
